# Impact of preoperative psychological support on outcomes in pancreatic cancer surgery: a propensity score-matched cohort study

**DOI:** 10.3389/fonc.2025.1631842

**Published:** 2025-10-24

**Authors:** Li Min, Suyan Jiang, Yanli Lu, Xinyu Zhang

**Affiliations:** ^1^ Third General Surgery Department, the People's Hospital of Lanzhou (Lanzhou Second Hospital Affiliated to Gansu University of Traditional Chinese Medicine), Lanzhou, Gansu, China; ^2^ First General Surgery Department, the Second People's Hospital of Lanzhou (Lanzhou Second Hospital Affiliated to Gansu University of Traditional Chinese Medicine), Lanzhou, Gansu, China

**Keywords:** pancreatic cancer, perioperative care, multidisciplinary psychological support, postoperative recovery, propensity score matching

## Abstract

**Background:**

Preoperative multidisciplinary psychological support (PMPS) has been associated with improved outcomes in several cancer populations, but its impact in pancreatic cancer remains underexplored.

**Method:**

We retrospectively reviewed 347 patients who underwent surgical treatment for pancreatic cancer between January 2020 and December 2022. Among them, 132 patients received preoperative multidisciplinary psychological support (PMPS), while 215 did not. To reduce confounding, 1:1 propensity score matching (PSM) was performed based on age, sex, comorbidities, tumor stage, and type of surgery, yielding 132 matched pairs (n = 264). The PMPS intervention included structured psychological counseling, perioperative education, relaxation techniques, and coordinated physical therapy. Primary outcomes were postoperative complication rate, length of hospital stay, and in-hospital mortality. Secondary outcomes included 30-day readmission, psychological status assessed by the Hospital Anxiety and Depression Scale (HADS), and patient satisfaction. Logistic regression and ROC analysis were conducted to evaluate the impact of PMPS.

**Results:**

Compared with the control group, patients in the PMPS group had a significantly lower incidence of postoperative complications (17.4% vs. 30.3%, P = 0.011), shorter hospital stay (10.0 ± 2.7 vs. 12.8 ± 3.3 days, P<0.001), and reduced in-hospital mortality (2.3% vs. 5.3%, P = 0.048). PMPS was associated with significantly improved postoperative anxiety and depression scores (P<0.001). Logistic regression indicated that PMPS independently reduced the risk of major complications (OR = 0.51, 95% CI: 0.30–0.88, P = 0.015). ROC curves demonstrated predictive value of PMPS for readmission (AUC = 0.725) and postoperative anxiety (AUC = 0.833).

**Conclusion:**

PMPS was associated with improved perioperative and psychological outcomes in patients undergoing pancreatic cancer surgery. Although this was a retrospective single-center study, our findings suggest that structured psychological support may have clinical value and should be considered as part of routine multidisciplinary care. Future multicenter prospective studies are warranted to validate these results.

## Introduction

Pancreatic cancer is a highly lethal malignancy, characterized by insidious onset, late-stage diagnosis, and poor prognosis (1, 2). Despite advances in surgical techniques and perioperative management, the postoperative complication rate remains high, and the physical and psychological burdens associated with major pancreatic surgery are considerable. Increasing evidence suggests that psychosocial distress—including anxiety, depression, and emotional instability—is common among patients undergoing treatment for pancreatic cancer and may negatively influence postoperative recovery, treatment adherence, and overall quality of life (3–5).

Surgical resection remains the cornerstone of curative therapy for pancreatic cancer, but it entails substantial physiological stress and complex recovery trajectories. Recent studies have highlighted that perioperative psychological states are independent predictors of surgical outcomes, including morbidity, immune function, and length of hospital stay (6–8). However, conventional perioperative care often underestimates the role of emotional and psychological support. Addressing this gap, perioperative multidisciplinary psychological support (PMPS)—including psychological counseling, cognitive-behavioral therapy, relaxation training, and social support—has emerged as a promising approach to mitigate psychological burden and improve overall recovery (9, 10).

Psychological distress, particularly anxiety and depression, has been shown to negatively influence surgical recovery by impairing immune function, delaying wound healing, and increasing the risk of postoperative complications (11). Recent meta-analyses in surgical oncology have confirmed that targeted psychological support can reduce perioperative morbidity and improve quality of life (12). Integrating preoperative multidisciplinary psychological support (PMPS) into standard care protocols for pancreatic cancer is therefore of particular importance, as it not only addresses the high psychological burden associated with this disease but also has the potential to enhance recovery, reduce complications, and optimize patient-centered outcomes in routine clinical practice. The PMPS model typically involves collaboration between surgeons, anesthesiologists, psychologists, psychiatrists, oncology nurses, and social workers. This integrative care framework is designed to provide individualized emotional support, stress coping strategies, and behavioral interventions throughout the surgical continuum—from preoperative preparation to postoperative rehabilitation (13–15). Although similar models have shown benefit in patients with other malignancies, such as breast and colorectal cancer (9, 10), there remains a paucity of high-quality evidence examining its effectiveness in pancreatic cancer patients, particularly during the perioperative period.

Preoperative psychological support has been shown to reduce anxiety, enhance coping mechanisms, and improve postoperative recovery, thereby contributing to better overall patient outcomes (16, 17). However, despite these benefits, psychological support in the preoperative period is often overlooked due to limited healthcare resources, time constraints in surgical settings, and the predominant focus on physical treatment rather than psychological well-being (18). Compared with breast or colorectal cancer, pancreatic cancer poses additional challenges for the delivery of preoperative psychological support (19). Patients with pancreatic cancer often experience greater psychological distress due to its poor prognosis and complex treatment regimens, making it more difficult to integrate structured psychological care during the preoperative period (20).

Furthermore, existing studies are limited by small sample sizes, heterogeneous interventions, and lack of rigorous comparison groups. To overcome these challenges, this study employs a retrospective cohort design with propensity score matching to evaluate the impact of PMPS on postoperative outcomes in pancreatic cancer patients undergoing surgery. We hypothesize that PMPS can reduce complication rates, shorten hospital stays, and improve psychological recovery. By investigating the real-world effect of PMPS within a multidisciplinary framework, this study aims to inform evidence-based integration of psychological care into surgical oncology protocols.

## Materials and methods

### Patient selection study design and participants:

This retrospective cohort study was conducted to assess the impact of perioperative multidisciplinary psychological support (PMPS) on postoperative recovery among pancreatic cancer patients. The study was carried out at the Department of Hepatobiliary Surgery of The Second People’s Hospital of Lanzhou between January 2020 and December 2022. Patients were eligible if they were 18 years or older, had histologically confirmed pancreatic ductal adenocarcinoma, and underwent curative-intent surgical procedures, including pancreaticoduodenectomy or distal pancreatectomy. Exclusion criteria included incomplete clinical or follow-up data, emergency surgery, documented psychiatric disorders or cognitive impairment, and in-hospital death within 24 hours of surgery. This study was designed and reported in accordance with the Strengthening the Reporting of Observational Studies in Epidemiology (STROBE) guidelines to ensure methodological transparency and reproducibility. Retrospective data were collected from electronic medical records. Institutional review board approval was obtained, and patients were informed of the study through public postings with an opt-out option available, in accordance with local ethical requirements.

Out of 347 eligible patients, 132 received PMPS, while 215 did not receive any structured psychological support. Patients who received only standard perioperative care formed the control group. To minimize confounding and ensure comparability, 1:1 propensity score matching (PSM) was performed, resulting in 132 matched pairs (264 patients) included in the final analysis.

### Perioperative multidisciplinary psychological support intervention

The perioperative multidisciplinary psychological support (PMPS) program was implemented as a structured intervention protocol designed to improve psychological resilience and enhance recovery outcomes in patients undergoing pancreatic cancer surgery. This program was delivered by a dedicated interdisciplinary team consisting of clinical psychologists, psychiatric nurses, attending surgeons, anesthesiologists, and licensed social workers. All members of the team received standardized training in psycho-oncology communication and patient-centered behavioral strategies prior to program rollout.

The intervention began in the preoperative phase, typically 3 to 7 days before surgery. Each patient underwent an initial psychological assessment using validated tools, including the Hospital Anxiety and Depression Scale (HADS) and the Perceived Stress Scale (PSS). Based on the results, individualized intervention plans were developed. Patients participated in two structured 30-minute counseling sessions focused on managing preoperative anxiety, clarifying misconceptions about the surgical procedure, building emotional resilience, and setting realistic recovery expectations. Cognitive-behavioral therapy (CBT) elements such as cognitive reframing, relaxation techniques, and goal-setting were integrated into the sessions. Patients also received written educational materials and were encouraged to maintain a preoperative stress journal.

During the postoperative hospitalization, psychological support was continued through 3 to 4 bedside intervention sessions, each lasting 15 to 30 minutes. These sessions aimed to address immediate emotional responses to surgery, including fear, helplessness, and uncertainty about prognosis. Techniques such as progressive muscle relaxation, guided imagery, and mindfulness breathing exercises were employed to mitigate stress and improve sleep quality. Nurses trained in supportive psychotherapy techniques facilitated daily check-ins to monitor emotional status, assess pain-coping capacity, and provide encouragement. Family members were actively involved through structured communication guidance and were encouraged to participate in bedside reassurance practices.

In addition to direct psychological care, the PMPS team collaborated with dietitians and physiotherapists to provide integrative support addressing patients’ overall quality of life, including nutritional confidence and early mobilization. For patients expressing significant emotional distress, psychiatric consultation and short-term pharmacologic support (e.g., low-dose anxiolytics) were provided on a case-by-case basis.

Physical therapy was provided to all patients as part of routine postoperative care. In the PMPS group, physical therapy was delivered in a structured and multidisciplinary manner as part of the comprehensive psychological support program, whereas in the control group it was delivered as standard care without coordinated psychological support. The control group received standard perioperative care, which included pain management, wound monitoring, nutritional counseling, early ambulation protocols, and discharge planning. However, they did not receive any structured psychological counseling, nor were they assessed or followed by the mental health team unless severe psychiatric symptoms emerged.

All PMPS interventions were documented in patient records, and fidelity to the intervention protocol was monitored weekly through interdisciplinary team meetings and random audits by the department’s quality control unit. Given the retrospective observational design, patient-level blinding was not feasible. However, outcome data were extracted by independent assessors who were not informed of group allocation to minimize bias. Intervention fidelity was monitored through weekly multidisciplinary team meetings and review of standardized session checklists to ensure consistency in the delivery of PMPS.

### Propensity score matching

To reduce selection bias and balance baseline characteristics between the two groups, propensity scores were estimated using a multivariable logistic regression model, with receipt of PMPS as the dependent variable. Covariates included in the model were age, sex, body mass index (BMI), ASA physical status classification, tumor stage, type of surgery, presence of comorbidities (e.g., diabetes, cardiovascular disease), and preoperative albumin and hemoglobin levels. Nearest-neighbor matching without replacement was applied, using a caliper width of 0.2 of the standard deviation of the logit of the propensity score. Covariate balance was evaluated using standardized mean differences (SMD), with an SMD < 0.1 indicating good balance.

### Outcome measures and data collection

Clinical data were extracted from the hospital’s electronic medical record system. The primary outcomes were postoperative complication rate (Clavien–Dindo grade ≥ II), length of hospital stay, and in-hospital mortality. Secondary outcomes included 30-day readmission rate, psychological status on postoperative day 7 measured by the Hospital Anxiety and Depression Scale (HADS), and patient satisfaction before discharge, based on a standardized satisfaction questionnaire. Postoperative complications were categorized into infectious, cardiopulmonary, thrombotic, gastrointestinal, and wound-related events. Long-term outcomes such as sustained recovery and quality of life were not assessed in this study. These will be an important focus of future prospective investigations.

### Statistical analysis

Descriptive statistics were used to summarize the demographic and clinical characteristics of the study population. Between-group differences were assessed using the chi-square test or Fisher’s exact test for categorical variables, and the Student’s t-test or Mann–Whitney U test for continuous variables, depending on data distribution. Logistic regression was used to identify independent predictors of postoperative complications and mortality. Receiver operating characteristic (ROC) curve analysis was performed to evaluate the discriminative value of PMPS in predicting readmission and psychological distress. All statistical analyses were performed using SPSS version 25.0 (IBM Corp., Armonk, NY, USA) and R version 4.0.5. A p-value < 0.05 was considered statistically significant. Sample size estimation was performed using PASS version 11.0. Sample size estimation was based on detecting a 20% relative reduction in postoperative complications with 80% power at a two-sided alpha of 0.05, yielding a minimum requirement of 120 patients per group.

## Results

### Baseline characteristics of pancreatic cancer patients with and without perioperative multidisciplinary psychological support

**Figure 1:**
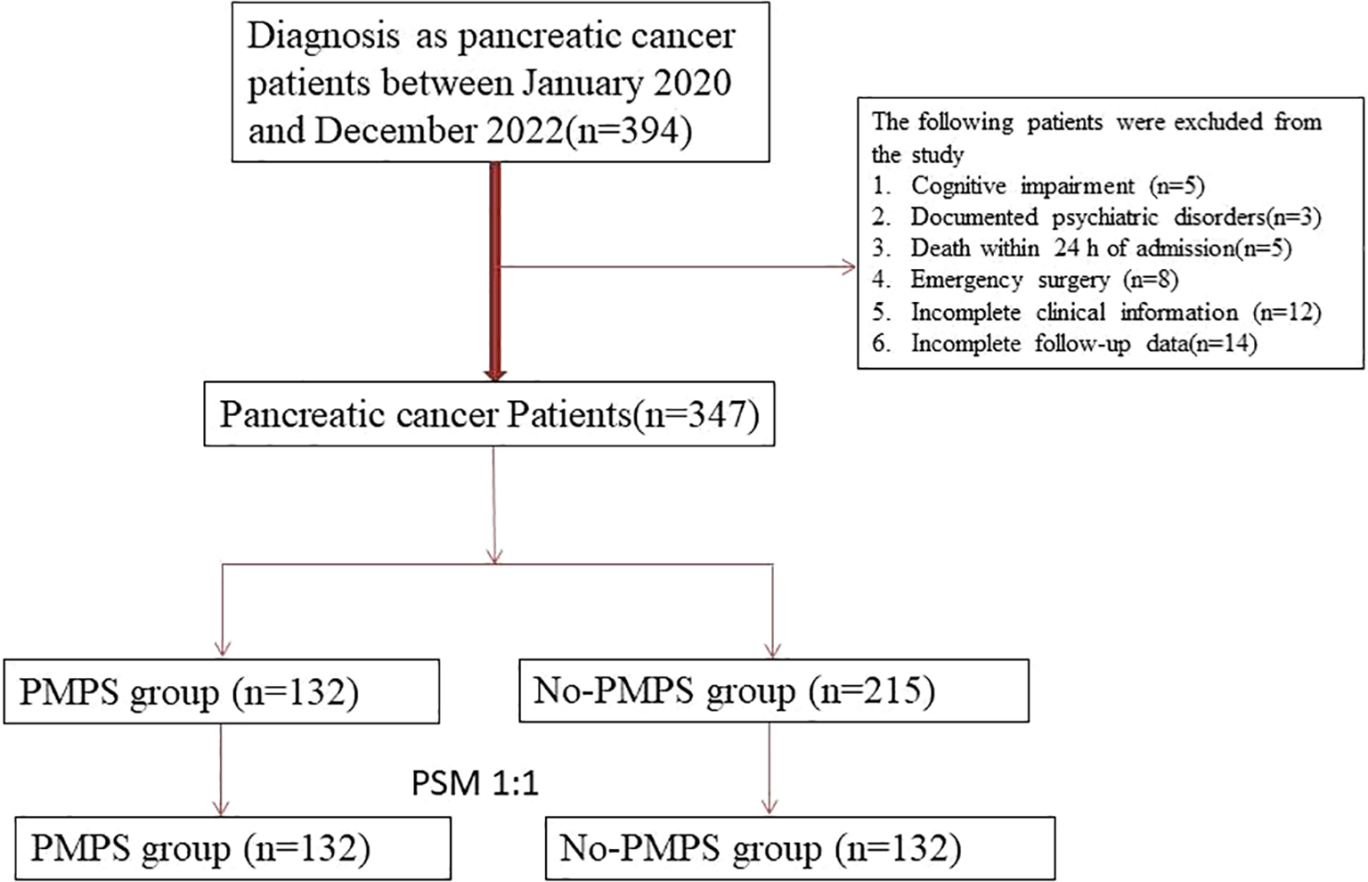
Flowchart of patient inclusion, exclusion, and 1:1 propensity score matching process in pancreatic cancer surgery cohort.

The patient selection process is shown in **Figure 1**. A total of 347 patients who underwent surgical treatment for pancreatic cancer were enrolled in this study. Of these, 132 patients received perioperative multidisciplinary psychological support (PMPS group), while 215 did not (control group). After 1:1 propensity score matching, 132 matched pairs (n = 264) were included in the final analysis.

Baseline characteristics before matching showed that patients in the PMPS group were slightly younger, had lower preoperative anxiety scores, and marginally better nutritional status. After matching, both groups were comparable in terms of age, sex, BMI, ASA score, tumor stage, type of surgery (pancreaticoduodenectomy vs. distal pancreatectomy), comorbidities (e.g., diabetes, cardiovascular disease), preoperative albumin and hemoglobin levels, and length of preoperative hospital stay (P > 0.05 for all; see **Table 1**).

**Table 1:** Baseline characteristics of patients with and without perioperative multidisciplinary psychological support (PMPS) after matching (n = 264).

Variable	PMPS group (n = 132)	Control group (n = 132)	P-value‡
Age, years	62 (55–69)	63 (56–70)	0.482
Sex, male	78 (59.1%)	75 (56.8%)	0.694
BMI (kg/m²)	22.6 ± 3.1	22.9 ± 3.2	0.372
ASA score ≥ III	49 (37.1%)	46 (34.8%)	0.689
Diabetes mellitus	41 (31.1%)	38 (28.8%)	0.713
Hypertension	53 (40.2%)	56 (42.4%)	0.735
Cardiovascular disease	22 (16.7%)	25 (18.9%)	0.625
Chronic pulmonary disease	9 (6.8%)	11 (8.3%)	0.645
Preoperative albumin (g/L)	39.5 ± 3.7	39.2 ± 4.0	0.498
Preoperative hemoglobin (g/L)	128 ± 13	126 ± 15	0.271
Preoperative HADS-Anxiety score	9.1 ± 2.6	9.3 ± 2.8	0.529
Tumor stage (TNM III–IV)	51 (38.6%)	48 (36.4%)	0.738
Type of surgery			
Pancreaticoduodenectomy	76 (57.6%)	74 (56.1%)	0.794
Distal pancreatectomy	56 (42.4%)	58 (43.9%)	
Operative time (min)	295 (270–330)	298 (275–340)	0.562
Estimated blood loss (mL)	320 (250–430)	335 (260–450)	0.371
Preoperative hospital stay (days)	4.5 ± 1.2	4.6 ± 1.4	0.627
Smoking history	38 (28.8%)	40 (30.3%)	0.783
Alcohol consumption	29 (22.0%)	27 (20.5%)	0.761
Family support (reported as adequate)	101 (76.5%)	96 (72.7%)	0.524

The values in parentheses are percentages unless indicated otherwise.

^‡^χ^2^ test or Fisher’s test.

mTBI, Mild Traumatic Brain Injury; GCS, Glasgow Coma Scale.

### Postoperative complications and clinical outcomes between groups

The incidence of postoperative complications was significantly lower in the PMPS group compared to the control group (17.4% vs. 30.3%, P = 0.011). The most notable reductions were observed in pulmonary infections (4.5% vs. 11.4%, P = 0.028), delayed gastric emptying (6.1% vs. 13.6%, P = 0.044), and postoperative delirium (2.3% vs. 8.3%, P = 0.037). No statistically significant differences were observed in rates of wound infection, pancreatic fistula, or deep vein thrombosis (P > 0.05).

Patients in the PMPS group had significantly shorter hospital stays (mean 10.0 ± 2.7 days vs. 12.8 ± 3.3 days, P < 0.001), lower in-hospital mortality (2.3% vs. 5.3%, P = 0.048), and reduced need for unplanned ICU admission (3.0% vs. 8.3%, P = 0.041). These results suggest that psychological support was associated with both physical and clinical improvements (see **Table 2**).

**Table 2:** Comparison of postoperative outcomes between PMPS group and control group (n = 264).

Variable	PMPS group (n = 132)	Control group (n = 132)	P-value‡
Postoperative complications (any)	23 (17.4%)	40 (30.3%)	0.011
Infectious complications	11 (8.3%)	21 (15.9%)	0.046
Pulmonary infection	6 (4.5%)	15 (11.4%)	0.028
Wound infection	3 (2.3%)	4 (3.0%)	0.702
Delayed gastric emptying	8 (6.1%)	18 (13.6%)	0.044
Postoperative delirium	3 (2.3%)	11 (8.3%)	0.037
Pancreatic fistula	5 (3.8%)	7 (5.3%)	0.547
Deep vein thrombosis	1 (0.8%)	3 (2.3%)	0.313
In-hospital mortality	3 (2.3%)	7 (5.3%)	0.048
Unplanned ICU admission	4 (3.0%)	11 (8.3%)	0.041
Length of hospital stay (days)	10.0 ± 2.7	12.8 ± 3.3	<0.001
30-day readmission	7 (5.3%)	16 (12.1%)	0.038
Psychological outcomes (Day 7)			
HADS-Anxiety score	6.1 ± 2.3	9.2 ± 2.9	<0.001
HADS-Depression score	5.7 ± 2.1	8.4 ± 3.0	<0.001
Patient satisfaction score (1–10 scale)	8.7 ± 0.9	7.4 ± 1.2	<0.001
Postoperative complications (any)	23 (17.4%)	40 (30.3%)	0.011

Values are presented as n (%) for categorical variables and mean ± SD for continuous variables.

‡P-values calculated using χ² test or Fisher’s exact test for categorical variables, and Student’s t-test for continuous variables.

PMPS, Perioperative Multidisciplinary Psychological Support; ICU, Intensive Care Unit; HADS, Hospital Anxiety and Depression Scale.

### Psychological and satisfaction outcomes

The PMPS group demonstrated significantly better psychological outcomes on postoperative day 7. Mean anxiety scores (HADS-A) were 6.1 ± 2.3 in the PMPS group vs. 9.2 ± 2.9 in the control group (P < 0.001), and depression scores (HADS-D) were 5.7 ± 2.1 vs. 8.4 ± 3.0, respectively (P < 0.001).

Moreover, patient satisfaction scores (rated on a 10-point scale at discharge) were markedly higher in the PMPS group (8.7 ± 0.9 vs. 7.4 ± 1.2, P < 0.001), with 94.7% of patients in the PMPS group reporting that psychological support had a “positive” or “very positive” effect on their recovery experience.

### Logistic regression analysis of postoperative complications

In univariate analysis, high ASA score (P = 0.024), low preoperative albumin (P = 0.016), and absence of PMPS (P = 0.006) were significantly associated with increased postoperative complications. Multivariate logistic regression confirmed PMPS as an independent protective factor (OR = 0.51, 95% CI: 0.30–0.88, P = 0.015), along with preoperative hypoalbuminemia (OR = 1.86, 95% CI: 1.09–3.17, P = 0.021) (see **Table 3**).

**Table 3:** Univariate and multivariate logistic regression analysis of risk factors associated with in-hospital mortality in mTBI patients.

Variable	Univariate analysis OR (95% CI)	P-value	Multivariate analysis OR (95% CI)	P-value
Age ≥ 65 years	1.22 (0.74–2.03)	0.436	–	–
Male sex	1.11 (0.66–1.86)	0.685	–	–
BMI ≥ 25 kg/m²	1.09 (0.63–1.89)	0.762	–	–
ASA score ≥ III	1.73 (1.06–2.83)	0.028*	1.48 (0.85–2.58)	0.166
Diabetes mellitus	1.31 (0.78–2.19)	0.310	–	–
Hypertension	1.20 (0.72–2.02)	0.484	–	–
Cardiovascular disease	1.39 (0.74–2.62)	0.305	–	–
Chronic pulmonary disease	1.46 (0.55–3.89)	0.456	–	–
Smoking history	1.41 (0.83–2.38)	0.205	–	–
Alcohol consumption	1.36 (0.78–2.38)	0.278	–	–
Preoperative albumin < 35 g/L	2.08 (1.18–3.68)	0.011*	1.89 (1.07–3.33)	0.029*
Preoperative hemoglobin < 110 g/L	1.43 (0.80–2.55)	0.225	–	–
HADS-A score ≥ 8 (moderate anxiety)	1.91 (1.12–3.28)	0.018*	1.61 (0.91–2.84)	0.098
HADS-D score ≥ 8 (moderate depression)	1.97 (1.13–3.42)	0.016*	1.64 (0.91–2.95)	0.097
TNM stage III–IV	1.29 (0.76–2.18)	0.344	–	–
Pancreaticoduodenectomy (vs distal)	1.47 (0.86–2.53)	0.162	–	–
Operative time ≥ 300 min	1.41 (0.83–2.41)	0.202	–	–
Estimated blood loss ≥ 400 mL	1.52 (0.89–2.60)	0.125	–	–
Preoperative hospital stay ≥ 5 days	1.18 (0.68–2.03)	0.555	–	–
Family support perceived as inadequate	1.86 (1.02–3.40)	0.043*	1.68 (0.89–3.17)	0.107
PMPS intervention (yes)	0.48 (0.28–0.84)	0.009	0.52 (0.29–0.91)	0.022

Variables with P < 0.05 in univariate analysis were included in multivariate regression.

*Statistically significant (P < 0.05).

OR, Odds Ratio; CI, Confidence Interval; ASA, American Society of Anesthesiologists; HADS, Hospital Anxiety and Depression Scale; PMPS, Perioperative Multidisciplinary Psychological Support.

### Effectiveness of PMPS on prognostic indicators

**Figure 2:**
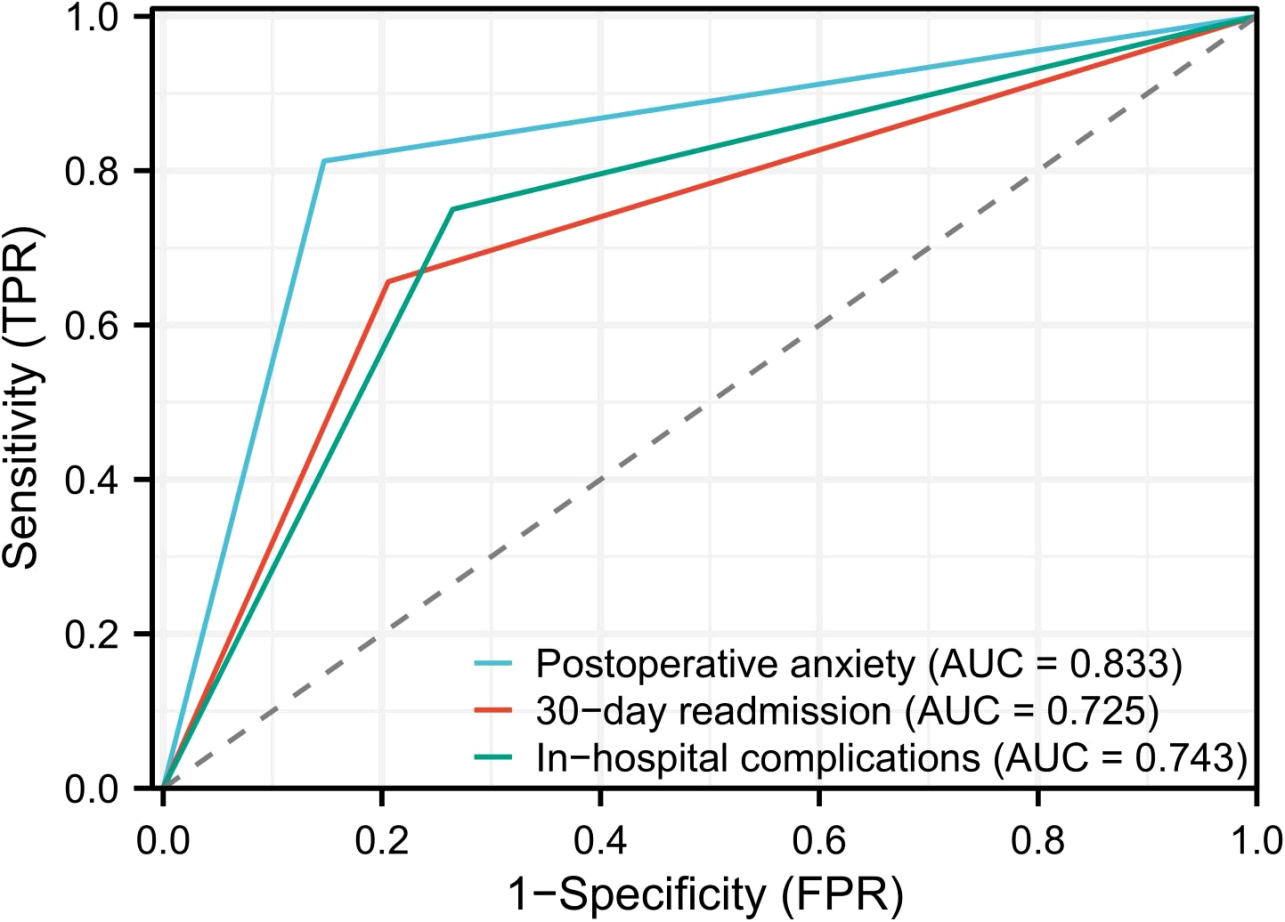
Receiver operating characteristic (ROC) curves demonstrating the predictive value of perioperative multidisciplinary psychological support for in-hospital complications, postoperative anxiety (HADS-A > 8), and 30-day readmission.

Compared with the control group, patients in the PMPS group had significantly lower rates of 30-day mortality (1.5% vs. 5.3%, P=0.048 ), 90 -day mortality (3.0% vs . 7.6%, P=0.049 ), and unplanned ICU admission (3.0% vs. 8.3%, P=0.041) (**Table 4**). Receiver operating characteristic (ROC) curves were used to evaluate the predictive utility of PMPS for key recovery endpoints. The area under the curve (AUC) was 0.833 for predicting postoperative anxiety (HADS-A > 8), 0.725 for 30-day readmission, and 0.743 for in-hospital complications (see **Figure 2**). These results indicate good predictive performance of PMPS for postoperative recovery.

**Table 4:** Comparison of clinical prognostic of TBI between integrated nursing intervention group and no integrated nursing intervention group (n = 246).

Outcome	PMPS group (n = 132)	Control group (n = 132)	P-value‡
30-day mortality	2 (1.5%)	7 (5.3%)	0.048
90-day mortality	4 (3.0%)	10 (7.6%)	0.049
Unplanned ICU admission	4 (3.0%)	11 (8.3%)	0.041

Values are presented as n (%) unless otherwise specified.

‡ P-values were calculated using χ² or Fisher’s exact test as appropriate.

PMPS, Perioperative Multidisciplinary Psychological Support; ICU, Intensive Care Unit.

## Discussion

Pancreatic cancer remains one of the most challenging malignancies to treat, with high rates of postoperative complications, prolonged recovery, and considerable psychological burden on patients (21, 22). Our study evaluated the impact of perioperative multidisciplinary psychological support (PMPS) on surgical outcomes in pancreatic cancer patients and demonstrated that such interventions were associated with improved recovery, reduced complication rates, and better psychological well-being. This study adds to the growing body of evidence supporting the role of psychological and psychosocial interventions in cancer care. We found that patients who received PMPS had significantly fewer postoperative complications, shorter hospital stays, and lower in-hospital mortality rates compared to those who did not receive psychological support.

These findings are consistent with previous studies showing that emotional distress, anxiety, and depression are associated with poorer surgical outcomes and delayed recovery in oncologic populations. For instance, Sebio-Garcia et al. and Tripuraneni et al. reported that addressing preoperative anxiety and improving psychological preparedness could enhance immune function and wound healing in surgical oncology settings (23, 24). In particular, the PMPS group in our study exhibited significantly lower rates of pulmonary complications and postoperative delirium, which are commonly associated with emotional stress, poor sleep, and impaired coping mechanisms. Our findings are aligned with those of studies in other cancer types, such as colorectal and breast cancer, where integrated psychological care has been shown to reduce postoperative morbidity.

Moreover, improved HADS scores and higher patient satisfaction in the PMPS group suggest that psychosocial care not only contributes to better physical outcomes but also plays a vital role in enhancing patients’ overall treatment experience. Another key result was the reduction in hospital stay by an average of 2.8 days in the PMPS group. Shorter hospital stays are associated with lower healthcare costs, fewer nosocomial complications, and better transitions to outpatient recovery. Similar benefits were reported by Tolvanen et al., who demonstrated that structured psychological support in surgical patients contributed to faster mobilization, improved nutritional intake, and earlier discharge (25, 26). Our data support this evidence, emphasizing that psychological interventions should be viewed as a core component of enhanced recovery pathways in pancreatic surgery. PMPS is inherently a multidisciplinary intervention. In our study, psychologists played a central role in providing emotional support, physical therapists promoted early mobilization and rehabilitation, and surgical nurses contributed to perioperative counseling and monitoring. This team-based approach ensured comprehensive care, reduced patient distress, and facilitated smoother recovery, underscoring the importance of multidisciplinary collaboration in complex surgical settings. Although this was a retrospective study, patient records and follow-up notes suggested that PMPS was generally well accepted. Patients appreciated the added psychological and rehabilitative support, while healthcare providers reported that the program improved communication, adherence, and perioperative cooperation. These findings indicate that PMPS is both feasible and acceptable in routine clinical practice.

Although our study focused on short-term postoperative outcomes, it is crucial to consider the long-term effects of PMPS on both recovery and quality of life. Extended follow-up is necessary to determine whether the psychological benefits observed in the perioperative period lead to sustained improvements in physical and mental health. Previous studies have shown that addressing preoperative anxiety and depression can not only improve immediate surgical outcomes but also lead to enhanced long-term coping and well-being (27, 28). Preoperative psychological interventions could reduce chronic stress, promote healthier immune responses, and potentially improve oncologic survival by enabling patients to better manage the psychosocial demands of cancer treatment (27, 28). As such, understanding the lasting impact of PMPS on patient quality of life and recovery trajectories should be a priority in future studies.

While our study was conducted in a single institution, it is important to recognize how different healthcare environments could affect the implementation of PMPS. Variations in healthcare resources, staffing, and institutional support structures can influence both the feasibility and scalability of such interventions. In settings with limited access to multidisciplinary teams, integrating PMPS may be challenging. However, our findings suggest that a collaborative approach, including psychologists, nurses, physical therapists, and other healthcare providers, can significantly improve patient outcomes. Additionally, cultural considerations play an important role in the success of psychological interventions. Patient receptiveness to psychological care can vary based on cultural norms and values, with some populations being less likely to seek or accept mental health support due to stigma or societal perceptions (29). Tailoring PMPS to fit these cultural differences, such as incorporating culturally relevant coping strategies and ensuring sensitivity to patient backgrounds, is essential for enhancing its effectiveness. Lastly, in resource-limited settings, alternative methods such as digital psychological support, telemedicine consultations, and community-based interventions could provide low-cost, scalable options for implementing PMPS, ensuring its accessibility and utility in diverse clinical environments.

However, our study is not without limitations. First, as a retrospective analysis, the ability to establish causal relationships is inherently limited. Although we used propensity score matching to control for known confounders, residual confounding may still exist. Second, the psychological intervention program, though standardized, was delivered by a single institution and may reflect resource-specific availability or cultural factors. Additionally, long-term psychological outcomes and oncologic survival were not assessed in this study, which could provide further insight into the enduring effects of PMPS. Future prospective, multicenter randomized controlled trials are necessary to validate these findings and determine the optimal content, duration, and delivery model of psychological support in surgical oncology. Furthermore, cost-effectiveness analyses would be valuable in supporting widespread implementation of PMPS programs, especially in resource-limited settings.

## Conclusion

In conclusion, this study demonstrates that perioperative multidisciplinary psychological support significantly improves postoperative outcomes in pancreatic cancer patients. Patients who received PMPS experienced fewer complications, shorter hospital stays, and better emotional recovery compared to those receiving standard care. These findings highlight the importance of integrating psychological support into perioperative protocols to enhance both physical and mental recovery. Our results provide compelling evidence for the adoption of structured psychosocial care as a standard component of surgical oncology pathways. Further research is warranted to optimize and standardize these interventions across institutions.

## Data Availability

The raw data supporting the conclusions of this article will be made available by the authors, without undue reservation.
